# Advancements in Rice Leaf Development Research

**DOI:** 10.3390/plants13060904

**Published:** 2024-03-21

**Authors:** Xiaoting Gong, Jian Chen, Yanxin Chen, Ying He, Dagang Jiang

**Affiliations:** College of Life Sciences, South China Agricultural University, Guangzhou 510642, China; gongxt2304@163.com (X.G.); 13424922617@stu.scau.edu.cn (J.C.); 19875909775@163.com (Y.C.); yinghe0916@163.com (Y.H.)

**Keywords:** leaf development, polarity establishment, hormones, rice

## Abstract

Rice leaf morphology is a pivotal component of the ideal plant architecture, significantly impacting rice yield. The process of leaf development unfolds through three distinct stages: the initiation of leaf primordia, the establishment and maintenance of polarity, and leaf expansion. Genes regulating leaf morphology encompass transcription factors, hormones, and miRNAs. An in-depth synthesis and categorization of genes associated with leaf development, particularly those successfully cloned, hold paramount importance in unraveling the complexity of rice leaf development. Furthermore, it provides valuable insights into the potential for molecular-level manipulation of rice leaf types. This comprehensive review consolidates the stages of rice leaf development, the genes involved, molecular regulatory pathways, and the influence of plant hormones. Its objective is to establish a foundational understanding of the creation of ideal rice leaf forms and their practical application in molecular breeding.

## 1. Introduction

Rice (*Oryza sativa*) is one of the most important cereal crops in the world and is also the staple food for nearly half of the world’s population [1]. However, the total area of arable land is limited; therefore, increasing grain yield per acre is an effective measure to alleviate population pressure and an important guarantee for national security [2]. Rice yield can be increased by enlarging the leaf area and enhancing the photosynthetic capacity of the population. The uppermost three leaves of rice, especially the flag leaf, are considered the main source of assimilates for photosynthesis and biomass synthesis [3,4]. Therefore, modifying the leaf shape of rice plays an important role in increasing yield [5].

Rice leaves are the primary organs for photosynthesis in plants, intricately linked with plant architecture and serving as a crucial indicator of morphological development [6]. They can be categorized into three types based on leaf position and role in overall plant development: basal leaves, transitional leaves, and cauline leaves. Basal leaves, situated near the base of the tiller nodes, provide nourishment for tillering, root development, and the differentiation of basal internodes, contributing to the formation of robust stems and large panicles. Transitional leaves play a pivotal role in root growth, stem elongation, spikelet primordium differentiation and development, and grain formation. Cauline leaves, comprising the uppermost three leaves, are instrumental during grain filling and maturation [4]. The size, thickness, drooping tendency, and leaf angle directly impact the leaf area index and canopy photosynthetic efficiency, thereby influencing rice yield [7].

Rice leaf development unfolds in three stages: the initiation of leaf primordia, the establishment and maintenance of polarity, and leaf expansion [8]. Leaves originate from the shoot apical meristem (SAM), where the central zone maintains the activity of stem cells, while the peripheral zone generates leaf primordia [9]. The development of leaves along the proximal–distal, adaxial–abaxial, and medial–lateral polarity axes determine the ultimate length, width, and curvature of mature rice leaves [10]. This process is intricately regulated by numerous functional genes (Table 1), transcription factors, and environmental factors, collectively shaping the effective photosynthetic surface area and photosynthetic efficiency [7,11]. In light of numerous studies on leaf development, this review synthesizes the complexities of leaf developmental processes, elucidating their underlying mechanisms [7,12,13]. Furthermore, we shed light on the intricate regulatory network governing rice leaf development, with a particular focus on the involvement of plant hormones at various developmental stages. Through this endeavor, we aspire to catalyze advancements in the development of ideal plant architecture and the pursuit of high-yield rice breeding.

## 2. Rice Leaf Development Process

**Figure 1:**
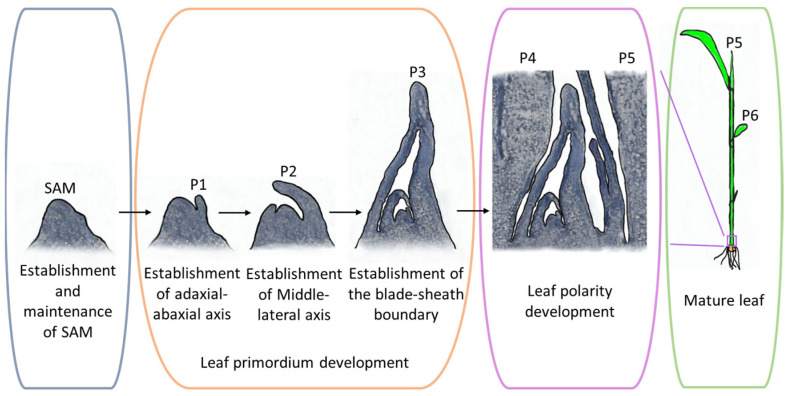
The development process of rice leaf. Leaf primordium cells are differentiated in the peripheral region of SAM, which rapidly divides into a crescent-shaped leaf primordium, denoted as P1. In this process, the adaxial–abaxial polarity of the leaf primordium is established. The process of establishing polarity along the medial–lateral axis occurs during the transition from the P1 to the P2 stage of leaf development. The P2 leaf primordium is hood shaped. At the highest of this hood, the vascular bundles of the leaf blade start to differentiate. At the same time, a younger leaf primordium arises on its opposite side. Subsequently, the older leaf primordium is transformed into an elongated conical configuration by cell division and cell elongation, marking the commencement of the P3 phase. It is during this phase that the boundary between the leaf and leaf sheath is delineated. The leaf elongates rapidly at the P4 stage, and the leaf sheath elongates at the P5 stage. Then, the tip of the leaf also emerges from the sheath. Finally, bulliform cells, stomatal cells, vascular bundles, etc., are differentiated and mature, and the whole leaf extends from the leaf sheath and flattens out. It marks the maturity of the leaves, termed P6. P1–P6 are used to divide the different stages of leaf primordium.

**Figure 2:**
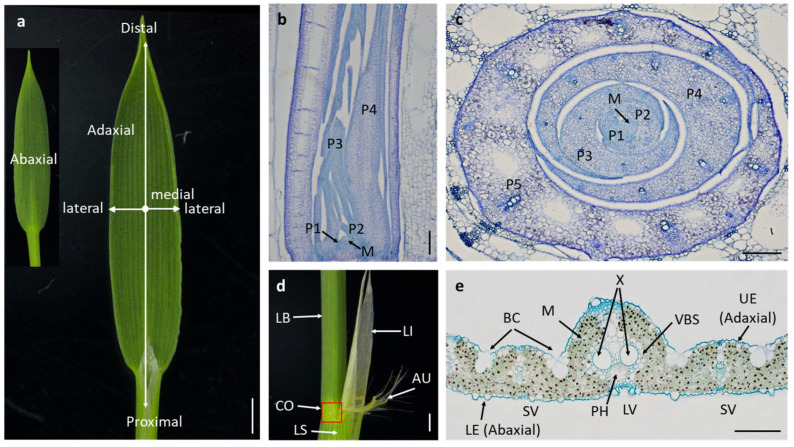
Rice leaf morphogenesis (a case study of Zhonghua 11). (**a**) Growth axis of the leaf. (**b**,**c**) Longitudinal section (**b**) and cross-section (**c**) of the seedling stem base area. (**d**) Boundary region between leaf blade and sheath. LB, leaf blade; LI, ligule; AU, auricle; CO, collar (indicated with a red square); LS, leaf sheath. (**e**) Cross-section of the middle of mature flag leaf. UE, upper epidermis; LE, lower epidermis; LV, large vascular bundle; SV, small vascular bundle; M, mesophyll; VBS, vascular bundle sheath; X, xylem; PH, phloem; BC, bulliform cell. P1–P6 are used to divide the different stages of leaf primordium; M, SAM. Scale, 1 mm (**a**,**d**), 100 μm (**b**,**c**,**e**).

The initiation of leaf development takes place in the peripheral region of the SAM [11,45] (Figure 1). When the apex of a leaf primordium extends beyond the SAM, the SAM’s base triggers the formation of another leaf primordium. The time interval between the emergence of two consecutive leaf primordia is known as the Plastochron (P) [46]. Initially, the peripheral zone of the SAM forms a small protuberance, growing toward the apex and the opposite side of the SAM, resulting in the formation of a crescent-shaped leaf primordium, denoted as P1 [47]. This primordium undergoes a transformation into a hood-like structure, termed P2, driven by rapid cell division and elongation at the apex and margin regions [11,45] (Figure 1). Subsequently, the leaf primordium’s two margins overlap, enclosing the SAM in an elongated conical configuration, marking the commencement of the P3 phase [12]. It is during this phase that the boundary between the leaf and leaf sheath is delineated [11,12]. Notably, epidermal cells engage in periclinal division, resulting in the formation of a distinctive protuberance at the leaf sheath’s base [7]. This phase also witnesses significant activities within various internal tissues. With the emergence of leaf auricles and ligules, the leaf primordium differentiates into young leaves and experiences significant elongation, signifying the onset of the P4 phase [48]. It is noteworthy that during the P1 to P4 stages, the elongation of the leaf sheath is temporarily suppressed. It is only after the leaf blade’s elongation culminates that the leaf sheath experiences rapid elongation, denoting the P5 stage [11] (Figure 1 and Figure 2a–d). This stage is succeeded by a sequence of events involving cell multiplication and differentiation, leading to the continuous expansion and thickening and ultimately culminating in the maturation of the leaf at the P6 stage [12,49] (Figure 1 and Figure 2a).

### 2.1. Initiation of Leaf Primordia

In the early phases of leaf development, cells located in the periphery of the SAM undergo a notable acceleration in their division, resulting in the formation of leaf primordia [5,45]. The initiation of these leaf primordia hinges on two intricately interconnected pathways: the Polar Auxin Transport pathway (PAT) and the Asymmetric Leaves1/Rough Sheath2/Phantastica (ARP)/Knotted1-like homeobox (KNOX) antagonistic pathway [50,51]. At the heart of this initiation process lies the need for a precise concentration of auxins to induce leaf primordia formation [45,52]. The PAT pathway is chiefly regulated by the auxin efflux carrier, PIN-FORMED1 (PIN1) proteins. PIN1 strategically localizes within the outermost epidermal cells of the meristematic tissue and serves as a conduit for the transport of auxins to the meristem’s apex [45]. The localized accumulation of auxin, driven by the PAT, dictates the specific location where leaf primordia sprout. Simultaneously, these newly formed leaf primordia consume auxins in their immediate vicinity, resulting in a distinct spacing between adjacent leaf primordia [50,53].

Within the ARP/KNOX pathway, antagonistic interactions between ARP and KNOX transcription factor families orchestrate the initiation of leaf primordia [54]. In the plant kingdom, KNOX transcription factors serve as key regulators of two critical aspects: the formation and sustenance of the SAM and the establishment of the proximal–distal axis within leaves [55]. Conversely, ARPs are primarily associated with cell differentiation processes occurring within leaf primordia [56]. These ARPs include *ASYMMETRIC LEAVES1* (*AS1*), *ROUGH SHEATH2* (*RS2*), and *PHANTASTICA* (*PHAN*). Within this intricate regulatory network, prominent KNOX family members, such as *SHOOT MERISTEMLESS* (*STM*) and *BREVIPEDICELLUS* (*BP*), exhibit downregulated expressions during the initiation of leaf primordia [57]. This downregulation is a fundamental prerequisite for subsequent cell or tissue differentiation to occur within the emerging leaf primordia. In rice, the SAM expresses *STM* and *BP* in a highly specific manner. *STM* suppresses the expression of *AS1* within the SAM [58]. Conversely, leaf primordia specifically express *AS1*, which, in turn, effectively inhibits *BP* expression. This intricate interaction ensures the smooth differentiation of peripheral region cells in the SAM [58,59]. It is important to note that high auxin concentrations may potentially repress the expressions of *BP* and *STM* within leaf primordia [60]. Consequently, a delicate balance of elevated auxin levels and upregulated *AS1* expression becomes a critical requirement for the initiation of leaf primordia. In summary, the ARP/KNOX antagonistic pathway offers a dynamic framework characterized by antagonistic interactions that not only facilitate the growth of leaf primordia but also maintain a lower degree of cell differentiation within the SAM’s peripheral region.

The *Oryza sativa homeobox* (*OSH*) gene in rice, a member of the KNOX family Class I, plays a crucial role in the formation and maintenance of the SAM. The expression of *KNOX* is positively regulated by cytokinins and the KNOX proteins themselves [59]. For instance, *OSH1* serves as a key regulator in SAM formation and maintenance by facilitating the catabolism of brassinosteroids (BRs) [14]. When *OSH1* expression is reduced, as observed in the *shoot organization* (*sho*) mutant during the early vegetative stage, the SAM becomes shorter and wider [15]. This alteration accelerates leaf initiation, resulting in a more randomized leaf sequence. Leaves in this context exhibit unique characteristics, appearing filamentous or shorter and narrower [15]. Another critical factor in this intricate dance is *phospholipase A1* (*PLA1*), a gene encoding the cytochrome P450 CYP78A11. *PLA1* likely plays a role in catalyzing substances that control leaf development [17]. It is expressed in leaf primordia and regulates the pace of leaf initiation and maturation [17]. In *pla1* mutants, the SAM undergoes enlargement due to increased cell division, leading to shortened intervals between leaf emergence. Consequently, plants with *pla1* mutations exhibit a significantly higher total leaf count, albeit with individual leaves being smaller and plant height reduced [17]. Therefore, *OSH* and *PLA1* are pivotal in preserving SAM functionality and ensuring the controlled development of leaf primordia.

Within *Arabidopsis thaliana*, the ERECTA family (ERf), including *ERECTA* (*ER*), *ERECTA-LIKE 1* (*ERL1*), and *ERL2*, have emerged as key players in the process of leaf initiation [61]. These genes are expressed throughout the entire process of the SAM and leaf primordia formation, exerting control over leaf initiation, SAM morphology development, inflorescence differentiation, and other critical processes [61]. Notably, when examining a triple mutant, *er erl1 erl2*, an intriguing phenomenon unfolds. The SAM in this mutant exhibits a distinctive appearance⁠—broader and flatter compared with that in the wild type [62]. This morphological alteration is accompanied by a noteworthy reduction in the expression of *PIN1* within leaf primordia [62]. Consequently, the rate of leaf initiation significantly slows down, leading to a decrease in the total leaf count [62]. In rice, the ERf comprises *ERECTA1* (*OsER1*), *OsER2*, and *OsERL*. While the knockout of *OsER1* and *OsER2* affects inflorescence development in rice [63,64], it is important to highlight that, as of the present, there is a noticeable gap in research concerning the involvement of ERf genes in regulating rice leaf development.

### 2.2. Establishment and Maintenance of Polarity

Once leaf characteristics are established, leaf primordial cells transition from simple cell division to a process of cell differentiation [7]. During this transformation, the leaf gradually develops three distinctive polarity axes: the proximal–distal, adaxial–abaxial, and medial–lateral axes [50,65,66] (Figure 2a). In rice, leaves are band shaped and the leaf sheath and leaf blade are bounded by the leaf collar, auricle, and ligule, which are located at the proximal axis and distal axis [67] (Figure 2a,d). The xylem, found nearer to the upper epidermis, defines the adaxial end, while the phloem, closer to the lower epidermis, characterizes the abaxial end. Between these two vascular bundles, bulliform cells are distributed [68] (Figure 2e). Significantly, the accurate establishment of polarity along the adaxial–abaxial axis serves as a prerequisite for the proper development of polarity along the proximal–distal and medial–lateral axes [51]. Mutations affecting polarity formation along the adaxial–abaxial axis can lead to abnormal leaf shapes, such as needle-like or rod-like leaves [10]. Therefore, the accurate development of three polarities is not only instrumental in shaping the leaf morphology but also serves as the foundational basis for normal leaf development.

#### 2.2.1. Establishment of the Adaxial–Abaxial Polarity

The adaxial–abaxial polarity defines the orientation from the upper epidermis to the lower epidermis within a leaf (Figure 2a). Correctly establishing polarity along this axis is crucial for enabling normal leaf development, resulting in a flat and expansive leaf structure while also influencing leaf thickness [50]. This polarity establishment coincides with the growth of the P1 leaf primordium toward the apex and the opposite side of the SAM [12] (Figure 1). In the context of leaf development, three genetic pathways are known to contribute to the establishment of polarity along the adaxial–abaxial axis: the homeodomain–leucine zipper (HD-ZIP) pathway, the AS1-AS2 pathway, and the small RNA pathway [50]. HD-ZIP III transcription factors play a pivotal role not only in the development of the SAM but also in shaping the adaxial face of lateral organs [10]. Signals originating from the SAM enhance the development of the adaxial face by activating HD-ZIP III, while the KANADI (KAN) and YABBY transcription factor families promote the development on the abaxial side [58]. Interestingly, HD-ZIP III and KAN function in an antagonistic manner to regulate adaxial–abaxial polarity in leaves [58]. In rice, members of the *HD-ZIP III* gene family, *OSHB1*, *OSHB3*, and *OSHB5*, participate in the initiation of leaf primordia and the development of polarity along the adaxial–abaxial axis, with their actions being dependent on auxins [10]. Additionally, *miRNA166* contributes to the establishment of adaxial–abaxial polarity by regulating the expression of *OSHB* genes [10]. Another gene, *OsHox32* (also referred to as *OSHB4*), influences leaf development by modulating the expression of *YABBY* genes [27]. The AS1-AS2 pathway inhibits the expression of *KAN*, thereby suppressing the development on the abaxial side, while promoting the adaxial face’s growth by enhancing the expression of *HD-ZIP III* [58,69]. *miRNA165/166* and trans-acting short-interfering RNA (ta-siRNA) exhibit opposite polarity distribution and jointly promote adaxial–abaxial polarity development through their antagonistic interplay [70]. *miRNA165/166* is primarily expressed at the abaxial face, contributing to abaxial face development by inhibiting the expression of *HD-ZIP III* genes, while ta-siRNA degrades auxin response factors (ARFs) on the abaxial side, thereby promoting abaxial face development [70,71]. *ARGONAUTE 1b* (*OsAGO1b*) suppresses the expressions of miRNA and ta-siRNA, resulting in a significant upregulation of *OSHB* and *OsARFs*, which are essential factors for controlling adaxial–abaxial axis polarity development [28]. These findings underscore the intricate interplay of these three regulatory pathways in shaping the adaxial–abaxial polarity during leaf development.

#### 2.2.2. Establishment of the Medial–Lateral Polarity

The medial–lateral polarity, which extends from a leaf’s midrib toward its sides, plays a vital role in determining leaf width [51] (Figure 2a). While research on the establishment of polarity along this axis remains relatively limited, a more comprehensive understanding has been gained regarding the development of leaf width following polarity establishment (Table 1). The process of establishing polarity along the medial–lateral axis occurs during the transition from the P1 to P2 stage of leaf development [12]. At these stages, the leaf primordium encircles the SAM as it grows along the medial–lateral axis, ultimately giving rise to a mature leaf with distinct central and lateral regions (Figure 1) [12]. Within the central region, the midrib serves as a sturdy structural element, providing support for the leaf and enabling efficient photosynthesis. This crucial midrib formation is orchestrated by genes belonging to the *YABBY* gene family, notably the *DROOPING LEAF* (*DL*) gene [22]. *DL* induces cell proliferation in the central leaf region, guiding midrib development. The midrib formation defects occur, causing the leaf drooping in the *DL* knockout plant [22]. The *WUSCHEL-related homeobox* (*WOX*) gene family, unique to plants, also contributes significantly to leaf development. For instance, *OsWOX3* participates in gibberellin (GA) synthesis and signaling pathway-associated negative feedback regulation, impacting both leaf lateral growth and the formation of vascular bundles [72]. Additionally, genes like *NARROW LEAF2* (*NAL2*) and *NAL3*, which are paralogs and encode transcriptional activators of *OsWOX3A*, play a crucial role in the development of leaf margin structures [23]. The suppression of these genes results in the absence of leaf margin structures and narrower leaves [23]. Within the *OsWOX3B* branch, the *LATERAL SYMMETRY1* (*LSY1*) gene shares a similar role in regulating leaf development along the medial–lateral axis [24]. *lsy1* mutants result in an incorrect regulation of this axis, leading to the loss of either or both lateral parts of leaves and, consequently, the emergence of asymmetric leaf phenotypes [24]. *OsWOX4* plays a pivotal role in regulating the maintenance of stem cell activity in the shoot meristem [73]. The downregulation of *OsWOX4* leads to reduced endogenous cytokinin synthesis, cessation of vascular bundle differentiation, midrib formation defects, and ultimately, narrower leaves [25]. In summary, the development of leaf margins and the midrib in rice is intricately regulated by *OsWOX3*, *OsWOX4*, and *DL* genes, collectively influencing leaf width and structure along the medial–lateral polarity.

#### 2.2.3. Establishment of the Proximal–Distal Polarity

The proximal–distal polarity, which determines the length of a leaf, refers to the direction from the leaf base to its tip [51,74] (Figure 2a). This polarity comprises key components, including the leaf sheath at the proximal end and the leaf blade at the distal end [65]. The leaf ligule, auricle, and collar collectively form the boundary between the leaf blade and the sheath [65] (Figure 2d). During the P3 stage, these boundary structures begin to take shape [12]. Notably, the epidermis near the proximal end undergoes radial cell division, facilitating the separation of the leaf blade from the sheath [12]. One essential gene in this process is *Oryza sativa LIGULELESS1* (*OsLG1*), which encodes a protein containing the SQUAMOSA promoter-binding protein (SBP) domain. *OsLG1* is primarily expressed in the collar region of young leaves [26]. *lg1* mutants result in the absence of collar, auricle, and ligule structures, causing the leaf blade to remain directly connected to the sheath [26]. This leads to a reduced leaf angle, impacting the leaf’s photosynthetic efficiency.

YABBY and WOX play significant roles in plant lateral organ formation and SAM function. *YAB3* RNA interference (RNAi) plants result in curled and knotted leaves, indicating their potential involvement in rice leaf cell differentiation [72]. The overexpression of *OsWOX3* can suppress *YAB3* expression, yielding defective leaf phenotypes similar to *YAB3* RNAi plants [72]. In rice, Class I KNOX members, including *OSH1*, *OSH6*, *OSH15*, *OSH43*, and *OSH71*, negatively regulate leaf development [59]. Their expressions are inhibited when leaf primordia initiate at the SAM’s edge. Ectopic expressions of these genes disrupt leaf development, causing abnormalities in the proximal–distal polarity development [59]. For instance, *osh1* mutants exhibit extended sheath regions or displacement of ligules and auricles and blurred SAM boundaries [14]. Overexpressing *OSH* genes can lead to ligule and auricle loss or even leaf blade absence, resembling phenotypes of *YAB3*-RNAi and *OsWOX3* overexpression plants [75]. Studies have revealed that *BOP1/2* genes in *Arabidopsis thaliana* express at the leaf proximal end and near the adaxial face, promoting the establishment of leaf proximal–distal polarity by inducing *AS2* and suppressing *KNOX* expression [76]. *Oryza sativa BLADE-ON-PETIOLE* (*OsBOP*) genes can activate the differentiation of proximal leaf sheaths and inhibit the differentiation of distal leaf blades. Triple mutants (*osbop1/2/3*) exhibit severe inhibition of sheath development, significantly larger leaf blades, and the absence of ligules and auricles [65]. While it is established that *OsBOP* genes regulate proximal leaf sheath development in rice, the precise regulatory mechanisms remain unclear. What is certain is that the genes of *OsLG1*, *YABBY*, *WOX*, and *KNOX* are involved in the polarity development of the proximal–distal axis.

### 2.3. Leaf Expansion

Leaf development advances once the three polarity axes are established, with leaf primordia undergoing further transformation through cell expansion and division, eventually maturing into fully developed leaves [49]. Critical to this process is the activity of the leaf’s intercalary meristem, which enables leaf extension. Epidermal cells, such as bulliform cells and stomatal cells, begin differentiation from the leaf tip and extend toward the base [7,77]. Simultaneously, vascular bundles mature, and sclerenchyma cells form outside these bundles [12]. These sclerenchyma cells and vascular bundles play pivotal roles in maintaining the flattened shape of rice leaves, and the development of sclerenchyma cells and bulliform cells can influence leaf curvature [68]. Upon the completion of leaf elongation, the leaf sheath undergoes rapid elongation. As the leaf tip emerges from the sheath, nearly all internal and epidermal leaf structures are fully formed, with the exception of the proximal region [12]. Throughout these intricate processes, numerous genes are instrumental in regulating leaf development (Table 1). For instance, genes like *Abaxially Curled Leaf 1* (*ACL1*) and *ACL2* influence leaf curvature by modulating the size of bulliform cells [78]. *OsHox32* overexpression impacts both the number and size of bulliform cells, resulting in leaf curling [27]. Mutations in *SHALLOT-LIKE1* (*SLL1*) and *Semi-Rolled Leaf2* (*SRL2*) lead to a reduction in the number of sclerenchyma cells on the abaxial surface, causing leaf rolling [29,30].

Aberrant cell division or cell elongation can also significantly affect leaf development. Growth-regulating factors (GRFs) and GRF-interacting factors (GIFs) collectively regulate rice leaf growth through precise control of cell multiplication [20]. *OsMBK3*, a member of the GIF family, positively regulates leaf size and internode elongation by influencing cell multiplication without altering cell size [21]. *STD1* and *NAL8* impact leaf size by regulating cell division and, consequently, cell numbers [32,33]. Additionally, *OsARF19* and *OsCCC1* influence leaf width by affecting cell size [34,35].

The plant’s vascular system, comprising the xylem and phloem, substantially influences leaf development. The counts of both large and small vascular bundles, as well as the spacing between the small vascular bundles, directly influence the width of rice leaves. Vascular bundle development is under the control of genetic factors and endogenous hormone levels [7]. Notably, *OsWOX3* and *OsWOX4* influence vascular bundle development by modulating GA and cytokinin levels, thus influencing leaf size [25,72]. The *NAL1* gene is primarily expressed in vascular tissues, regulating leaf size by influencing cell division, vascular bundle count, and PAT [31]. Overexpressing *NAL2* and *NAL3* results in wider leaves with increased vascular bundles, in contrast to the phenotype observed in *nal2/3* transgenic antisense plants [23]. Lastly, the *NAL9* gene encodes an ATP-dependent Clp protease proteolytic subunit and displays a narrow leaf phenotype throughout the entire growth period in its mutants [38]. Overall, leaf development is a complex process that is regulated by many genes. Abnormalities in either gene may lead to leaf defects.

## 3. Plant Hormones and Leaf Development

### 3.1. Auxins

Auxins, crucial plant hormones, play a pivotal role in orchestrating the initiation and growth of leaves within the SAM. They finely regulate these processes by affecting cell growth and division in the early stages of leaf development [51]. Auxins at high concentrations can suppress *KNOX* gene expression, thereby triggering the formation of leaf primordia [51,79]. The direction of auxin movement is facilitated by PIN proteins, which enable polar transport. When auxin inhibitors are applied, PIN protein activity is disrupted, halting leaf development [80]. Conversely, the exogenous application of auxins to the SAM can reactivate and stimulate leaf development [80]. During leaf primordium formation, auxins are transported from the adaxial face of the leaf primordium toward the SAM. This movement maintains a low auxin concentration at the adaxial face, ensuring the proper establishment and maintenance of leaf polarity [81].

Two key genes, *NAL7* and *NAL1*, influence leaf width by modulating auxin biosynthesis and its PAT [31,39]. *NAL7* primarily governs leaf width by regulating auxin biosynthesis. Compared with wide types, *nal7* mutants exhibit decreased auxin levels in the leaf base, resulting in narrower leaves [39]. In contrast, *nal1* mutants display reduced capacity for PAT, leading to fewer veins and narrower leaves [31]. Studies have shown that *OsCR4*, a receptor-like kinase, contributes to auxin distribution and concentration gradients. Loss of *OsCR4* function results in an aberrant distribution of auxin within leaves and has severe effects on vascular tissue formation and epidermal development. This leads to the formation of wrinkled, smaller leaves [40]. Additionally, auxins may coordinate the development of the proximal–distal polarity and adaxial–abaxial polarity in leaves after the initiation of leaf primordia. High auxin concentrations affect distal leaf development and the formation of the vascular system [13]. Disruptions in auxin transport or signaling pathways can lead to a shift in leaf development toward the abaxial end [13]. However, recent research suggests that both adaxial and abaxial leaf surfaces perceive auxin similarly, indicating a more intricate regulatory network for auxins in leaf development [82].

### 3.2. Brassinosteroids (BRs)

Brassinosteroids play a vital role in SAM maintenance and collar formation by activating the expression of genes related to cell elongation and cell wall modification [14,51]. *OSH1* suppresses the BR content by inhibiting the signaling pathway and accelerating its inactivation to ensure the formation and maintenance of leaf primordia [14]. In *osh1* mutants and *CYP734A* RNAi plants, the BR catabolism pathway is inhibited. It results in premature cell differentiation, tissue hardening, boundary defects between leaf and sheath, and SAM and leaf primordium [14].

In rice, exogenous BR application influences the division and elongation of cells located at the junction between the leaf blade and leaf sheath at the adaxial face [83]. This asymmetrical cell development between the collar’s adaxial and abaxial sides results in the curvature of the collar and an increased leaf angle [83]. This phenomenon underscores the intimate correlations of BR biosynthesis and signaling with leaf angle. The biosynthesis of BR primarily involves enzymes from the cytochrome P450 protein family. Genes responsible for encoding these enzymes have a direct impact on leaf angle regulation. Notable examples include *OsDWARF11/D11*, *OsDWARF4*, *CYP90D2/D2*, and *OsCYP51G3* [84,85,86,87]. Mutations in these genes hinder the elongation of parenchyma cells near the collar, leading to upright leaves [84,85,86,87]. The BR signaling pathway involves receptor kinases, such as *OsBRI1* and *OsBZR1*. Knockout of *OsBRI1* and *OsBZR1* disrupts the elongation of parenchyma cells near the lamina joint, causing leaves to be upright [88,89]. *OsBSK3* directly interacts with *OsBRI1* and positively regulates BR signaling [90]. Therefore, the biosynthesis and signaling pathways of BR exert a notable influence on the development of parenchyma cells located at the adaxial face of the leaf collar, consequently acting as positive regulators of the leaf angle in rice.

### 3.3. Cytokinins

The development of leaves, from initiation to full morphogenesis, is intricately tied to the delicate balance between auxins and cytokinins [91]. Cytokinins, in particular, play a pivotal role in the establishment and maintenance of the SAM [92]. *LONELY GUY* (*LOG*), responsible for activating cytokinins, is critical; its loss prematurely halts SAM development [16]. On the contrary, increased cytokinin levels enhance SAM activity, profoundly influencing leaf development and the formation of floral meristems [64]. Furthermore, the cytokinin oxidase/dehydrogenase (OsCKX) genes, including *OsCKX1*, *OsCKX2*, and *OsCKX8* as suppressors, negatively regulate basal internode diameter, leaf dimensions, and grain size [93]. However, *OsCKX4* as a promotor positively influences these several characters [93]. Notably, *OsDOF11* directly regulates *OsCKX4* expression levels, thus affecting cytokinin levels, and a loss of *OsDOF11* function results in smaller leaves and reduced plant height [42].

### 3.4. Gibberellins (GAs)

GAs play a crucial role in the intricate process of rice leaf development. GAs primarily regulate cell differentiation and elongation [41]. Any mutations in genes associated with the GA signaling transduction can significantly impact the size of plant organs [41]. Throughout the course of leaf development, increased GA level correlates with both larger cell size and cell number in leaves, resulting in enlargement leaves [94]. Conversely, decreased GA level or the plant’s weaker sensitivity to GAs leads to smaller leaves and dwarfed plants [94]. In this context, the rice *PLA1* and *PLA2* genes come into play downstream in the GA signaling pathway, where they contribute to the regulation of leaf maturation [18]. They also participate actively in the process of rice leaf elongation mediated by GAs, primarily by influencing cell proliferation [18]. Furthermore, *OsGA2ox6* plays a pivotal role by facilitating the breakdown of GAs. This action reduces the endogenous GA levels, resulting in the upregulation of *OSH1* and *TB1* expressions [41]. The net effect is stunted plant growth, increased tillering number, and smaller leaves [41]. Moreover, *GIBBERELLIN INSENSITIVE DWARF1* (*GID1*) and *GID2* exert control over plant height and leaf size by modulating the GA pathway [95,96]. The intricate interplay between high cytokinin concentrations and low GA levels serves to maintain the undifferentiated state of stem cells in the SAM [97]. This delicate balance effectively inhibits the initiation of leaves [97]. *KNOX* genes play a dual role in this process. They positively regulate cytokinin biosynthesis and simultaneously negatively regulate GA synthesis by inhibiting *GA20ox* activity [79]. This dual action ensures that the SAM’s form and function are maintained as they should be. Therefore, gibberellin is involved in the regulation of leaf initiation and leaf size.

In conclusion, the interactions between plant hormones, such as auxins, cytokinins, and BRs, as well as their homeostasis and spatial signal transmission, can effectively control and maintain the dynamic balance of cell division and differentiation in the SAM and influence the leaf development process.

## 4. Prospects

The morphology of rice leaves encompasses various vital attributes, including length, width, thickness, curvature, and angle, collectively comprising the blade, collar, auricle, ligule, and sheath. As the primary site for photosynthesis, rice leaf morphology holds a profound sway over plant growth and development and, ultimately, yield [7]. Therefore, the optimization of leaf morphology becomes a pivotal undertaking in breeding high-yield rice varieties with an ideal plant architecture. For an ideal rice plant, the top three leaves must ideally manifest characteristics of being lengthy, erect, slender, V-shaped, and robust in thickness. Length and erectness contribute to expanded leaf surface area, while slender leaves economize on space utilization. V-shaped leaves retain their tautness and are resistant to drooping, and thicker leaves manifest vigorous photosynthesis with reduced senescence, ultimately amplifying the overall exploitation of sunlight [6]. These favorable leaf traits denote the plant’s capacity to generate more carbohydrates, a cardinal prerequisite for achieving high yields. Hence, the crux of realizing ideal plant types lies in the synthesis of these favorable leaf traits, emphasizing the pressing need for deeper insights into rice leaf development processes and the intricate web of regulatory mechanisms.

The development of rice leaves stands as a multifaceted process, meticulously governed by numerous genes (Table 1), intricately intertwined with various hormones, and susceptible to the influence of environmental factors. Despite considerable strides in unraveling the regulatory mechanisms underpinning rice leaf development, much of this research remains compartmentalized. A comprehensive understanding of the intricate network of interactions among different genes involved in rice leaf development, alongside the delicate interplay between genes, hormones, and environmental cues, remains somewhat elusive. Furthermore, the knowledge landscape of rice leaf development is unevenly contoured, with a predominant focus on genes regulating leaf width, while leaf thickness has been relegated to relative obscurity. This inequity can be attributed, in part, to the inherent intricacy in quantifying leaf thickness characteristics. Yet, with the advent of advanced technologies, such as genome-wide association studies (GWAS) and single-cell sequencing, prospects loom large for a more lucid delineation of the regulatory network governing leaf development. Concurrently, the continued evolution and refinement of gene-editing technologies augur well for the sculpting and refinement of ideal leaf morphologies. These advancements are poised to significantly elevate the molecular design technology framework for breeding ideal rice plant types, ultimately ushering in substantial enhancements in rice yields.

## Figures and Tables

**Table 1:** List of genes that regulate rice leaf development.

Gene Name	Gene ID	Effect on Leaf Development	Reference
*OSH1*	LOC_Os03g51690	Maintain the function and shape of SAM, affect blade–sheath boundary development	[14]
*SHO1*	LOC_Os04g43050	Maintain the function and shape of SAM, involved in leaf initiation and size	[15]
*LOG*	LOC_Os01g40630	Involved in SAM development	[16]
*PLA1*	LOC_Os10g26340	Maintain the function and shape of SAM, involved in leaf initiation and size	[17]
*PAL2*	LOC_Os01g68000	Regulate leaf growth via cell multiplication	[18]
*OsARF18*	LOC_Os06g47150	Affect the size and number of bulliform cells, involved in the number of epidermic cells and auxin signal	[19]
*OsGRF1*	LOC_Os02g53690	Regulate leaf size by cell multiplication	[20]
*OsMBK3*	LOC_Os03g52320	Regulate leaf size by cell multiplication	[21]
*DL*	LOC_Os03g11600	Involved in midrib development	[22]
*NAL2*	LOC_Os11g01130	Regulate vascular pattern, leaf size, and marginal region	[23]
*NAL3*	LOC_Os12g01120	Regulate vascular pattern, leaf size, and marginal region	[23]
*LSY1*	LOC_Os05g02730	Involved in the development of leaf shape and size	[24]
*OsWOX4*	LOC_Os04g55590	Control vascular development and leaf width	[25]
*OsLG1*	LOC_Os04g56170	Affect blade–sheath boundary development	[26]
*OsHox32*	LOC_Os03g43930	Affect the size and number of bulliform cells and leaf size	[27]
*OsAGO1b*	LOC_Os04g47870	Involved in adaxial sclerenchyma cell development	[28]
*SLL1*	LOC_Os09g23200	Regulate abaxial sclerenchyma cell number	[29]
*SRL2*	LOC_Os03g19520	Regulate abaxial sclerenchyma cell number and leaf width	[30]
*NAL1*	LOC_Os04g52479	Control cell division, number of vascular bundles, and auxin polar transport	[31]
*STD1*	LOC_Os02g56540	Control cell division and leaf size	[32]
*NAL8*	LOC_Os07g15880	Control cell division and leaf width	[33]
*OsARF19*	LOC_Os06g48950	Regulate cell size and leaf width	[34]
*OsCCC1*	LOC_Os08g23440	Regulate cell size and leaf width	[35]
*OsEXPA8*	LOC_Os01g14650	Increase leaf number and size by cell expansion	[36]
*OsGASR1*	LOC_Os03g55290	Increase cell and leaf length	[37]
*NAL9*	LOC_Os03g29810	Control leaf width	[38]
*NLA7*	LOC_Os03g06654	Involved in leaf shape and auxin biosynthesis	[39]
*OsCR4*	LOC_Os03g43670	Leaf development mediated by auxin	[40]
*OsGA2ox6*	LOC_Os04g44150	Regulate the catabolism of gibberellin and leaf size	[41]
*OsDOF11*	LOC_Os02g47810	Control leaf size	[42]
*WL1*	LOC_Os03g57240	Involved in vascular pattern and leaf width	[5]
*NRL1*	LOC_Os12g36890	Affect leaf shape and bulliform cell development	[43]
*DGL1*	LOC_Os01g49000	Regulate leaf length and number	[44]

## Data Availability

No data were used for the research described in the article.

## References

[1] Mohapatra P.K., Sahu B.B. (2022). Importance of rice as human food. Panicle Architecture of Rice and Its Relationship with Grain Filling.

[2] He P., Wang X., Zhang X., Jiang Y., Tian W., Zhang X., Li Y., Sun Y., Xie J., Ni J. (2018). Short and narrow flag leaf1, a GATA zinc finger domain-containing protein, regulates flag leaf size in rice (*Oryza sativa*). BMC Plant Biol..

[3] Jebbouj R., Brahim E.Y. (2009). Barley yield losses due to defoliation of upper three leaves either healthy or infected at boot stage by *Pyrenophora teres* f. *teres*. Eur. J. Plant Pathol..

[4] Zhai L., Yan A., Shao K., Wang S., Wang Y., Chen Z.H., Xu J. (2023). *Large Vascular Bundle Phloem Area 4* enhances grain yield and quality in rice via source-sink-flow. Plant Physiol..

[5] You J., Xiao W., Zhou Y., Shen W., Ye L., Yu P., Yu G., Duan Q., Zhang X., He Z. (2022). The APC/C^TAD1^-WIDE LEAF 1-NARROW LEAF 1 pathway controls leaf width in rice. Plant Cell.

[6] Yuan L. (2017). Progress in super-hybrid rice breeding. Crop J..

[7] Wang J., Xu J., Qian Q., Zhang G. (2020). Development of rice leaves: How histocytes modulate leaf polarity establishment. Rice Sci..

[8] Wang H., Kong F., Zhou C. (2021). From genes to networks: The genetic control of leaf development. J. Integr. Plant Biol..

[9] Shen W., Sun J., Xiao Z., Feng P., Zhang T., He G., Sang X. (2023). Narrow and stripe leaf 2 regulates leaf width by modulating cell cycle progression in rice. Rice.

[10] Itoh J., Hibara K., Sato Y., Nagato Y. (2008). Developmental role and auxin responsiveness of Class III homeodomain leucine zipper gene family members in rice. Plant Physiol..

[11] Miya M., Yoshikawa T., Sato Y., Itoh J.I. (2021). Genome-wide analysis of spatiotemporal expression patterns during rice leaf development. BMC Genom..

[12] Itoh J., Nonomura K., Ikeda K., Yamaki S., Inukai Y., Yamagishi H., Kitano H., Nagato Y. (2005). Rice plant development: From zygote to spikelet. Plant Cell Physiol..

[13] Satterlee J.W., Scanlon M.J. (2019). Coordination of leaf development across developmental axes. Plants.

[14] Tsuda K., Kurata N., Ohyanagi H., Hake S. (2014). Genome-wide study of *KNOX* regulatory network reveals brassinosteroid catabolic genes important for shoot meristem function in rice. Plant Cell.

[15] Itoh J.I., Kitano H., Matsuoka M., Nagato Y. (2000). *Shoot organization* genes regulate shoot apical meristem organization and the pattern of leaf primordium initiation in rice. Plant Cell.

[16] Kurakawa T., Ueda N., Maekawa M., Kobayashi K., Kojima M., Nagato Y., Sakakibara H., Kyozuka J. (2007). Direct control of shoot meristem activity by a cytokinin-activating enzyme. Nature.

[17] Miyoshi K., Ahn B.O., Kawakatsu T., Ito Y., Itoh J., Nagato Y., Kurata N. (2004). *PLASTOCHRON1*, a timekeeper of leaf initiation in rice, encodes cytochrome P450. Proc. Natl. Acad. Sci. USA.

[18] Mimura M., Itoh J. (2014). Genetic interaction between rice *PLASTOCHRON* genes and the gibberellin pathway in leaf development. Rice.

[19] Huang J., Li Z., Zhao D. (2016). Deregulation of the osmir160 target gene *OsARF18* causes growth and developmental defects with an alteration of auxin signaling in rice. Sci. Rep..

[20] Lu Y., Meng Y., Zeng J., Luo Y., Feng Z., Bian L., Gao S. (2020). Coordination between *GROWTH-REGULATING FACTOR1* and *GRF-INTERACTING FACTOR1* plays a key role in regulating leaf growth in rice. BMC Plant Biol..

[21] Shimano S., Hibara K.I., Furuya T., Arimura S.I., Tsukaya H., Itoh J.I. (2018). Conserved functional control, but distinct regulation, of cell proliferation in rice and *Arabidopsis* leaves revealed by comparative analysis of *GRF-INTERACTING FACTOR 1* orthologs. Development.

[22] Yamaguchi T., Nagasawa N., Kawasaki S., Matsuoka M., Nagato Y., Hirano H.Y. (2004). The *YABBY* gene *DROOPING LEAF* regulates carpel specification and midrib development in *Oryza sativa*. Plant Cell.

[23] Ishiwata A., Ozawa M., Nagasaki H., Kato M., Noda Y., Yamaguchi T., Nosaka M., Shimizu-Sato S., Nagasaki A., Maekawa M. (2013). Two WUSCHEL-related homeobox genes, *narrow leaf2* and *narrow leaf3*, control leaf width in rice. Plant Cell Physiol..

[24] Honda E., Yew C.L., Yoshikawa T., Sato Y., Hibara K.I., Itoh J.I. (2018). *LEAF LATERAL SYMMETRY1*, a member of the *WUSCHEL-RELATED HOMEOBOX3* gene family, regulates lateral organ development differentially from other paralogs, *NARROW LEAF2* and *NARROW LEAF3* in rice. Plant Cell Physiol..

[25] Yasui Y., Ohmori Y., Takebayashi Y., Sakakibara H., Hirano H.Y. (2018). *WUSCHEL-RELATED HOMEOBOX4* acts as a key regulator in early leaf development in rice. PLoS Genet..

[26] Lee J., Park J.J., Kim S.L., Yim J., An G. (2007). Mutations in the rice *liguleless* gene result in a complete loss of the auricle, ligule, and laminar joint. Plant Mol. Biol..

[27] Li Y.Y., Shen A., Xiong W., Sun Q.L., Luo Q., Song T., Li Z.L., Luan W.J. (2016). Overexpression of *OsHox32* results in pleiotropic effects on plant type architecture and leaf development in rice. Rice.

[28] Li Y., Yang Y., Liu Y., Li D., Zhao Y., Li Z., Liu Y., Jiang D., Li J., Zhou H. (2019). Overexpression of *OsAGO1b* induces adaxially rolled leaves by affecting leaf abaxial sclerenchymatous cell development in rice. Rice.

[29] Zhang G.H., Xu Q., Zhu X.D., Qian Q., Xue H.W. (2009). *SHALLOT-LIKE1* is a KANADI transcription factor that modulates rice leaf rolling by regulating leaf abaxial cell development. Plant Cell.

[30] Liu X., Li M., Liu K., Tang D., Sun M., Li Y., Shen Y., Du G., Cheng Z. (2016). *Semi-Rolled Leaf2* modulates rice leaf rolling by regulating abaxial side cell differentiation. J. Exp. Bot..

[31] Jiang D., Fang J., Lou L., Zhao J., Yuan S., Yin L., Sun W., Peng L., Guo B., Li X. (2015). Characterization of a null allelic mutant of the rice *NAL1* gene reveals its role in regulating cell division. PLoS ONE.

[32] Fang J., Yuan S., Li C., Jiang D., Zhao L., Peng L., Zhao J., Zhang W., Li X. (2018). Reduction of ATPase activity in the rice kinesin protein *Stemless Dwarf 1* inhibits cell division and organ development. Plant J..

[33] Chen K., Guo T., Li X.M., Yang Y.B., Dong N.Q., Shi C.L., Ye W.W., Shan J.X., Lin H.X. (2019). *NAL8* encodes a prohibitin that contributes to leaf and spikelet development by regulating mitochondria and chloroplasts stability in rice. BMC Plant Biol..

[34] Zhang S., Wang S., Xu Y., Yu C., Shen C., Qian Q., Geisler M., Jiang D.A., Qi Y. (2015). The auxin response factor, *OsARF19*, controls rice leaf angles through positively regulating *OsGH3-5* and *OsBRI1*. Plant Cell Environ..

[35] Chen Z.C., Yamaji N., Fujii-Kashino M., Ma J.F. (2016). A cation-chloride cotransporter gene is required for cell elongation and osmoregulation in rice. Plant Physiol..

[36] Ma N., Wang Y., Qiu S., Kang Z., Che S., Wang G., Huang J. (2013). Overexpression of *OsEXPA8*, a root-specific gene, improves rice growth and root system architecture by facilitating cell extension. PLoS ONE.

[37] Lee S.C., Kim S.J., Han S.K., An G., Kim S.R. (2017). A gibberellin-stimulated transcript, *OsGASR1*, controls seedling growth and α-amylase expression in rice. J. Plant Physiol..

[38] Li W., Wu C., Hu G., Xing L., Qian W., Si H., Sun Z., Wang X., Fu Y., Liu W. (2013). Characterization and fine mapping of a novel rice narrow leaf mutant *nal9*. J. Integr. Plant Biol..

[39] Fujino K., Matsuda Y., Ozawa K., Nishimura T., Koshiba T., Fraaije M.W., Sekiguchi H. (2008). *NARROW LEAF 7* controls leaf shape mediated by auxin in rice. Mol. Genet. Genom. MGG.

[40] Wang J., Yan L.L., Yue Z.L., Li H.Y., Ji X.J., Pu C.X., Sun Y. (2020). Receptor-like kinase *OsCR4* controls leaf morphogenesis and embryogenesis by fixing the distribution of auxin in rice. J. Genet. Genom..

[41] Lo S.F., Yang S.Y., Chen K.T., Hsing Y.I., Zeevaart J.A., Chen L.J., Yu S.M. (2008). A novel class of *gibberellin 2-oxidases* control semidwarfism, tillering, and root development in rice. Plant Cell.

[42] Wu Y., Wang L., Ansah E.O., Peng W., Zhang W., Li P., An G., Xiong F. (2022). The sucrose transport regulator *OsDOF11* mediates cytokinin degradation during rice development. Plant Physiol..

[43] Hu J., Zhu L., Zeng D., Gao Z., Guo L., Fang Y., Zhang G., Dong G., Yan M., Liu J. (2010). Identification and characterization of *NARROW AND ROLLED LEAF 1*, a novel gene regulating leaf morphology and plant architecture in rice. Plant Mol. Biol..

[44] Komorisono M., Ueguchi-Tanaka M., Aichi I., Hasegawa Y., Ashikari M., Kitano H., Matsuoka M., Sazuka T. (2005). Analysis of the rice mutant dwarf and gladius leaf 1. Aberrant katanin-mediated microtubule organization causes up-regulation of gibberellin biosynthetic genes independently of gibberellin signaling. Plant Physiol..

[45] Krishnamurthy K.V., Bahadur B., John Adams S., Venkatasubramanian P., Bahadur B., Venkat Rajam M., Sahijram L., Krishnamurthy K.V. (2015). Origin, development and differentiation of leaves. Plant Biology and Biotechnology: Volume I: Plant Diversity, Organization, Function and Improvement.

[46] van Campen J.C., Yaapar M.N., Narawatthana S., Lehmeier C., Wanchana S., Thakur V., Chater C., Kelly S., Rolfe S.A., Quick W.P. (2016). Combined chlorophyll fluorescence and transcriptomic analysis identifies the P3/P4 transition as a key stage in rice leaf photosynthetic development. Plant Physiol..

[47] Shaaf S., Bretani G., Biswas A., Fontana I.M., Rossini L. (2019). Genetics of barley tiller and leaf development. J. Integr. Plant Biol..

[48] Kusumi K., Chono Y., Shimada H., Gotoh E., Tsuyama M., Iba K. (2010). Chloroplast biogenesis during the early stage of leaf development in rice. Plant Biotechnol..

[49] Gonzalez N., Vanhaeren H., Inzé D. (2012). Leaf size control: Complex coordination of cell division and expansion. Trends Plant Sci..

[50] Nakayama H., Leichty A.R., Sinha N.R. (2022). Molecular mechanisms underlying leaf development, morphological diversification, and beyond. Plant Cell.

[51] Lv Z., Zhao W., Kong S., Li L., Lin S. (2023). Overview of molecular mechanisms of plant leaf development: A systematic review. Front. Plant Sci..

[52] Scarpella E., Barkoulas M., Tsiantis M. (2010). Control of leaf and vein development by auxin. Cold Spring Harb. Perspect. Biol..

[53] Fleming A.J. (2005). The control of leaf development. New Phytol..

[54] Hay A., Tsiantis M. (2010). KNOX genes: Versatile regulators of plant development and diversity. Development.

[55] Hay A., Tsiantis M. (2009). A KNOX family TALE. Curr. Opin. Plant Biol..

[56] Byrne M.E., Simorowski J., Martienssen R.A. (2002). *ASYMMETRIC LEAVES1* reveals knox gene redundancy in *Arabidopsis*. Development.

[57] Nishimura A., Ito M., Kamiya N., Sato Y., Matsuoka M. (2002). *OsPNH1* regulates leaf development and maintenance of the shoot apical meristem in rice. Plant J..

[58] Hasson A., Blein T., Laufs P. (2010). Leaving the meristem behind: The genetic and molecular control of leaf patterning and morphogenesis. Comptes Rendus Biol..

[59] Tsuda K., Ito Y., Sato Y., Kurata N. (2011). Positive autoregulation of a *KNOX* gene is essential for shoot apical meristem maintenance in rice. Plant Cell.

[60] Heisler M.G., Ohno C., Das P., Sieber P., Reddy G.V., Long J.A., Meyerowitz E.M. (2005). Patterns of auxin transport and gene expression during primordium development revealed by live imaging of the *Arabidopsis* inflorescence meristem. Curr. Biol..

[61] Kosentka P.Z., Overholt A., Maradiaga R., Mitoubsi O., Shpak E.D. (2019). EPFL signals in the boundary region of the SAM restrict its size and promote leaf initiation. Plant Physiol..

[62] Chen M.K., Wilson R.L., Palme K., Ditengou F.A., Shpak E.D. (2013). ERECTA family genes regulate auxin transport in the shoot apical meristem and forming leaf primordia. Plant Physiol..

[63] Zhang Y., Li S., Xue S., Yang S., Huang J., Wang L. (2018). Phylogenetic and CRISPR/Cas9 studies in deciphering the evolutionary trajectory and phenotypic impacts of rice ERECTA genes. Front. Plant Sci..

[64] Guo T., Lu Z.Q., Shan J.X., Ye W.W., Dong N.Q., Lin H.X. (2020). *ERECTA1* acts upstream of the *OsMKKK10*-*OsMKK4*-*OsMPK6* cascade to control spikelet number by regulating cytokinin metabolism in rice. Plant Cell.

[65] Toriba T., Tokunaga H., Shiga T., Nie F., Naramoto S., Honda E., Tanaka K., Taji T., Itoh J.I., Kyozuka J. (2019). *BLADE-ON-PETIOLE* genes temporally and developmentally regulate the sheath to blade ratio of rice leaves. Nat. Commun..

[66] Xiong Y., Jiao Y. (2019). The diverse roles of auxin in regulating leaf development. Plants.

[67] Mohapatra P.K., Sahu B.B. (2022). Botany of rice plant. Panicle Architecture of Rice and Its Relationship with Grain Filling.

[68] Xu P., Ali A., Han B., Wu X. (2018). Current advances in molecular basis and mechanisms regulating leaf morphology in rice. Front. Plant Sci..

[69] Husbands A.Y., Benkovics A.H., Nogueira F.T., Lodha M., Timmermans M.C. (2015). The *ASYMMETRIC LEAVES* complex employs multiple modes of regulation to affect adaxial-abaxial patterning and leaf complexity. Plant Cell.

[70] Nogueira F.T., Madi S., Chitwood D.H., Juarez M.T., Timmermans M.C. (2007). Two small regulatory RNAs establish opposing fates of a developmental axis. Genes Dev..

[71] Fouracre J.P., Poethig R.S. (2016). The role of small RNAs in vegetative shoot development. Curr. Opin. Plant Biol..

[72] Dai M., Hu Y., Zhao Y., Liu H., Zhou D.X. (2007). A *WUSCHEL-LIKE HOMEOBOX* gene represses a *YABBY* gene expression required for rice leaf development. Plant Physiol..

[73] Ohmori Y., Tanaka W., Kojima M., Sakakibara H., Hirano H.Y. (2013). *WUSCHEL-RELATED HOMEOBOX4* is involved in meristem maintenance and is negatively regulated by the *CLE* gene *FCP1* in rice. Plant Cell.

[74] Xiong Y., Wu B., Du F., Guo X., Tian C., Hu J., Lü S., Long M., Zhang L., Wang Y. (2021). A crosstalk between auxin and brassinosteroid regulates leaf shape by modulating growth anisotropy. Mol. Plant.

[75] Sentoku N., Sato Y., Matsuoka M. (2000). Overexpression of rice *OSH* genes induces ectopic shoots on leaf sheaths of transgenic rice plants. Dev. Biol..

[76] Jun J.H., Ha C.M., Fletcher J.C. (2010). *BLADE-ON-PETIOLE1* coordinates organ determinacy and axial polarity in *Arabidopsis* by directly activating *ASYMMETRIC LEAVES2*. Plant Cell.

[77] Conklin P.A., Strable J., Li S., Scanlon M.J. (2019). On the mechanisms of development in monocot and eudicot leaves. New Phytol..

[78] Li L., Shi Z.Y., Li L., Shen G.Z., Wang X.Q., An L.S., Zhang J.L. (2010). Overexpression of *ACL1* (*abaxially curled leaf 1*) increased Bulliform cells and induced Abaxial curling of leaf blades in rice. Mol. Plant.

[79] Kalve S., De Vos D., Beemster G.T. (2014). Leaf development: A cellular perspective. Front. Plant Sci..

[80] Scanlon M.J. (2003). The polar auxin transport inhibitor N-1-naphthylphthalamic acid disrupts leaf initiation, KNOX protein regulation, and formation of leaf margins in maize. Plant Physiol..

[81] Qi J., Wang Y., Yu T., Cunha A., Wu B., Vernoux T., Meyerowitz E., Jiao Y. (2014). Auxin depletion from leaf primordia contributes to organ patterning. Proc. Natl. Acad. Sci. USA.

[82] Bhatia N., Åhl H., Jönsson H., Heisler M.G. (2019). Quantitative analysis of auxin sensing in leaf primordia argues against proposed role in regulating leaf dorsoventrality. eLife.

[83] Tong H., Chu C. (2018). Functional specificities of brassinosteroid and potential utilization for crop improvement. Trends Plant Sci..

[84] Li H., Jiang L., Youn J.H., Sun W., Cheng Z., Jin T., Ma X., Guo X., Wang J., Zhang X. (2013). A comprehensive genetic study reveals a crucial role of *CYP90D2/D2* in regulating plant architecture in rice (*Oryza sativa*). New Phytol..

[85] Mori M., Nomura T., Ooka H., Ishizaka M., Yokota T., Sugimoto K., Okabe K., Kajiwara H., Satoh K., Yamamoto K. (2002). Isolation and characterization of a rice dwarf mutant with a defect in brassinosteroid biosynthesis. Plant Physiol..

[86] Sakamoto T., Morinaka Y., Ohnishi T., Sunohara H., Fujioka S., Ueguchi-Tanaka M., Mizutani M., Sakata K., Takatsuto S., Yoshida S. (2006). Erect leaves caused by brassinosteroid deficiency increase biomass production and grain yield in rice. Nat. Biotechnol..

[87] Xia K., Ou X., Tang H., Wang R., Wu P., Jia Y., Wei X., Xu X., Kang S.H., Kim S.K. (2015). Rice microRNA osa-miR1848 targets the obtusifoliol 14α-demethylase gene *OsCYP51G3* and mediates the biosynthesis of phytosterols and brassinosteroids during development and in response to stress. New Phytol..

[88] Bai M., Zhang L., Gampala S.S., Zhu S., Song W., Chong K., Wang Z. (2007). Functions of OsBZR1 and 14-3-3 proteins in brassinosteroid signaling in rice. Proc. Natl. Acad. Sci. USA.

[89] Zhao J., Wu C., Yuan S., Yin L., Sun W., Zhao Q., Zhao B., Li X. (2013). Kinase activity of *OsBRI1* is essential for brassinosteroids to regulate rice growth and development. Plant Sci..

[90] Zhang B., Wang X., Zhao Z., Wang R., Huang X., Zhu Y., Yuan L., Wang Y., Xu X., Burlingame A.L. (2016). *OsBRI1* activates BR signaling by preventing binding between the TPR and kinase domains of *OsBSK3* via phosphorylation. Plant Physiol..

[91] Wu W., Du K., Kang X., Wei H. (2021). The diverse roles of cytokinins in regulating leaf development. Hortic. Res..

[92] Hussain S., Nanda S., Zhang J., Rehmani M.I.A., Suleman M., Li G., Hou H. (2021). Auxin and cytokinin interplay during leaf morphogenesis and phyllotaxy. Plants.

[93] Rong C., Liu Y., Chang Z., Liu Z., Ding Y., Ding C. (2022). Cytokinin oxidase/dehydrogenase family genes exhibit functional divergence and overlap in rice growth and development, especially in control of tillering. J. Exp. Bot..

[94] Gonzalez N., De Bodt S., Sulpice R., Jikumaru Y., Chae E., Dhondt S., Van Daele T., De Milde L., Weigel D., Kamiya Y. (2010). Increased leaf size: Different means to an end. Plant Physiol..

[95] Sasaki A., Itoh H., Gomi K., Ueguchi-Tanaka M., Ishiyama K., Kobayashi M., Jeong D.H., An G., Kitano H., Ashikari M. (2003). Accumulation of phosphorylated repressor for gibberellin signaling in an F-box mutant. Science.

[96] Ueguchi-Tanaka M., Ashikari M., Nakajima M., Itoh H., Katoh E., Kobayashi M., Chow T.Y., Hsing Y.I., Kitano H., Yamaguchi I. (2005). *GIBBERELLIN INSENSITIVE DWARF1* encodes a soluble receptor for gibberellin. Nature.

[97] Gordon S.P., Chickarmane V.S., Ohno C., Meyerowitz E.M. (2009). Multiple feedback loops through cytokinin signaling control stem cell number within the *Arabidopsis* shoot meristem. Proc. Natl. Acad. Sci. USA.

